# Chiral Drugs: An Overview

**Published:** 2006-06

**Authors:** Lien Ai Nguyen, Hua He, Chuong Pham-Huy

**Affiliations:** 1 *Department of Pharmacy, Lucile Salter Packard Children’s Hospital, Stanford University Medical Center, 725 Welch Road, Palo Alto, USA;*; 2 *Department of Analytical Chemistry, China Pharmaceutical University, Nanjing, China;*; 3 *Laboratory of Toxicology, Faculty of Pharmacy, University of Paris 5, 4 avenue de l’Observatoire, Paris, France*

**Keywords:** analysis, chiral drugs, chiral separation, chiral terms, enantioselective antibodies, metabolism, pharmacokinetics, pharmacology, toxicology

## Abstract

About more than half of the drugs currently in use are chiral compounds and near 90% of the last ones are marketed as racemates consisting of an equimolar mixture of two enantiomers. Although they have the same chemical structure, most isomers of chiral drugs exhibit marked differences in biological activities such as pharmacology, toxicology, pharmacokinetics, metabolism etc. Some mechanisms of these properties are also explained. Therefore, it is important to promote the chiral separation and analysis of racemic drugs in pharmaceutical industry as well as in clinic in order to eliminate the unwanted isomer from the preparation and to find an optimal treatment and a right therapeutic control for the patient. In this article, we review the nomenclature, pharmacology, toxicology, pharmacokinetics, metabolism etc of some usual chiral drugs as well as their mechanisms. Different techniques used for the chiral separation in pharmaceutical industry as well as in clinical analyses are also examined.

## INTRODUCTION

Chiral chemistry was discovered by Louis Pasteur, a French chemist and biologist, when he separated by hand for the first time, in 1848, the two isomers of sodium ammonium tartrate (1, 2). However, it needed about a century later to find that the phenomenon of chirality plays a key role not only in the life of plants and animals but also in pharmaceutical, agricultural and other chemical industries. All proteins, enzymes, amino acids, carbohydrates, nucleosides and a number of alkaloids and hormones are chiral compounds. In pharmaceutical industries, 56% of the drugs currently in use are chiral products and 88% of the last ones are marketed as racemates consisting of an equimolar mixture of two enantiomers (3-5). In contrast to chiral artificial products, all natural compounds are under single enantiomeric form, for example, all natural amino acids are l-isomer (levorotatory) as well as all natural sugars (carbohydrates) are d-isomer (dextrorotatory). Although they have the same chemical structure, most enantiomers of racemic drugs exhibit marked differences in biological activities such as pharmacology, toxicology, pharmacokinetics, metabolism etc. The mechanisms of chiral drugs with biological environment are now explained. Therefore, it is important to promote the chiral separation and analysis of racemic drugs in pharmaceutical industry as well as in clinic in order to eliminate the unwanted isomer from the preparation and to find an optimal treatment and a right therapeutic control for the patient.

Chirality is now a top-class subject for academic research as well as for pharmaceutical development. Accounting for the important role of chiral separation, the 2001 Nobel Prize in Chemistry has been awarded to three scientists: Dr. William S. Knowles and Pr. K. Barry Sharpless in USA and Pr. Ryori Nyori in Japan, for their development of asymmetric synthesis using chiral catalysts in the production of single enantiomer drugs or chemicals (6, 7). Thanks to a wide range of new technologies for chiral separation, US Food and Drug Administration (FDA) recently recommends the assessments of each enantiomer activity for racemic drugs in body and promotes the development of new chiral drugs as single enantiomers (7).

In this article, we review the nomenclature of chiral compounds, the biological activities such as pharmacology, toxicology, pharmacokinetics, metabolism etc of some usual racemic drugs in therapeutics as well as their mechanisms. Different techniques used for the chiral separation in pharmaceutical industry as well as in clinical analyses are also examined.

## NOMENCLATURE OF CHIRAL COMPOUNDS

The terminology used to describe different stereochemical properties is resumed as following (8-10).

Chirality (also sometimes called stereoisomerism or enantiomerism or dissymmetry) is a property of an object which is non-superimposable with its mirror image. The origin of the word chiral is Greek *cheir*, which means ‘handedness’. When a molecule cannot be superimposed on its mirror image, this molecule and its image are called chiral. It is like left and right hands. The two non-superimposable mirror-image forms of chiral molecules are called enantiomers. Enantiomers are most commonly formed when a carbon atom contains four different substituents (asymmetric carbon atom or stereogenic carbon or also called chiral center). A chiral molecule is a molecule having at least one asymmetric carbon. Carbon is not the only atom that can act as an asymmetric center. Sulfur, phosphorus and nitrogen can sometimes form chiral molecules such as omeprazole, cyclophosphamide and methaqualone, respectively. Chiral molecules exhibit optical activity, so enantiomers are also sometimes called optical isomers. The two enantiomers of such compounds may be classified as levorotary (l-isomer) or dextrorotary (d-isomer) depending on whether they rotate plane-polarized light in a left (-) or right (+) -handed manner, respectively. An equimolar mixture (50/50) of the two enantiomers of a chiral compound is called a racemic mixture (racemate) with sign (±) or (d, l) that does not exhibit optical activity. Optical isomers or enantiomers are molecules having the same chemical formula, the same physical and chemical properties, but differing in their optical activity and their spatial arrangement.

**Figure 1:**
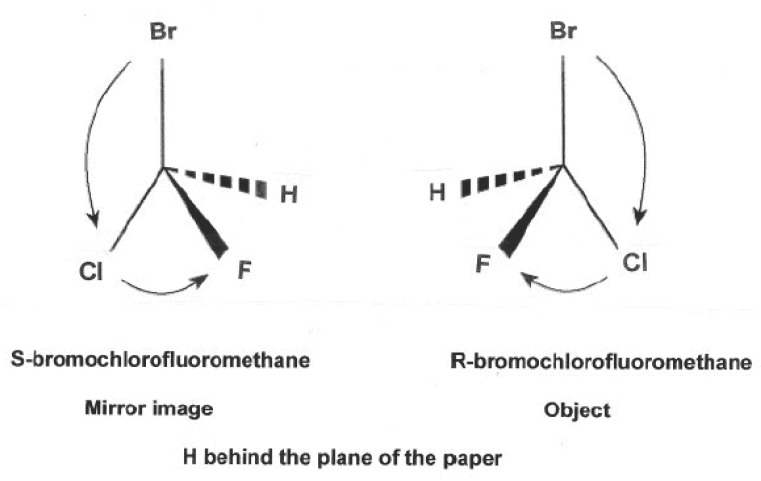
H behind the plane of the paper.

Enantiomers are now determined by their spatial arrangement (3 dimensions) of substituents (groups) around a chiral center (asymmetric carbon) in the molecule. This configuration follows the Cahn-Ingold-Prelog (CIP) convention (11) which is used to assign priorities to substituent groups. This system is based on a set of rules for ordering the substituents attached to the asymmetric atom by using sequence rules to assign priorities. There are different rules, being the simplest: “substituents of the higher atomic number precede those with lower ones”. First, draw a picture so that the atom with lowest priority, for example H, seems below the plane of the picture (Fig. 1). Then start counting from the highest priority (highest atomic number or highest mass) to the lowest one, for example ^35^Br→^17^Cl →^9^F or for another example Cl→CH_2_CH_3_→ CH_3_. If the counting goes in a clockwise direction, the configuration is designated as R (rectus or right); otherwise in a counter clockwise direction, it is S (sinister or left). A racemate is designated as R,S. Each R- and S-enantiomers can rotate plane-polarized light, therefore they can be designated as R(+) or R (-) and S (+) or S(-). The optical activity of an enantiomer is determined by a polarimeter or by optical rotary dispersion and circular dichroism. The spatial arrangement of a chiral compound is determined by nuclear magnetic resonance or/and X-ray crystallography diffraction. The R/S tridimensional configuration allows to explain the interaction of enantiomers with their biologic receptors.

Diastereomers are any molecules which have two or more chiral centers. A diastereomer with two chiral carbon has four isomers. Unlike enantiomers, the physical and chemical properties of diastereomers can differ and consequently, their chemical characterization is easy and their biological activities are often different. This is the basis for derivatization of enantiomers to form diastereomers in chiral separation and also for the explanation of enantiomer activities with their chiral receptors in the body. Diastereomers, enantiomers and geometric isomers form a family called stereoisomers that are molecules having the same chemical formulas but differing only with respect to the spatial arrangement.

Eutomer refers to bioactive enantiomer or enantiomer having higher pharmacological activity. Its opposite is called distomer.

Epimers are two diastereoisomers having a different configuration at only one chiral centre.

Enantioselectivity is a property of a process whereby one enantiomer is expressed exclusively or predominantly over the other. In pharmacological terms, that means a biological structure (enzyme, antibody or receptor) which exhibits affinity towards one enantiomer over the other.

Enantioselective assay is an analytical method capable of separating and quantifying enantiomers.

In a stereoselective synthesis, one of a set of isomers is predominantly or exclusively formed whereas in a stereospecific synthesis, one isomer leads to one product while another isomer leads to the opposite product.

Homochirality is the biological chirality in which all biologic compounds have the same chirality such as all amino acids are levorotary isomers.

Chiral switch is a procedure used to transform an old racemic drug into its single active enantiomer. This new enantiomeric drug developed by a pharmaceutical manufacturer will receive additional patent protection and a new generic name.

## PHARMACOLOGY

The body with its numerous homochiral compounds being amazingly chiral selector, will interact with each racemic drug differently and metabolize each enantiomer by a separate pathway to generate different pharmacological activity. Thus, one isomer may produce the desired therapeutic activities, while the other may be inactive or, in worst cases, produce undesired or toxic effects (9, 12, 13, 15, 25, 32).

In pharmacology area, only racemic drugs will be examined and their activity can be divided into three main groups. The majority of racemic pharmaceuticals have one major bioactive enantiomer (called eutomer), the other is inactive or less active (distomer) or toxic or can exert other desired or undesired pharmacological properties. The second category is intended to drugs where the two enantiomers are equally active and have the same pharmacodynamics. The last one is racemic drugs having only one eutomer, but the distomer could be transformed in body into its bioactive antipode by chiral inversion (12-15).

### Group 1. Racemic drugs with one major bioactive enantiomer

In this group, there are a number of cardiovascular drugs, agents widely used for the treatment of hypertension, heart failure, arrhythmias, and other diseases. Among these are the β-adrenergic blocking agents, calcium channel antagonists and angiotensin-converting enzyme (ACE) inhibitors.

Levorotary–isomer of all β-blockers is more potent in blocking β-adrenoceptors than their dextrorotary-isomer, such as S(-)-propranolol is 100 times more active than its R(+)-antipode (16-18). A number of β-blockers are still marketed as racemic form such as acebutolol, atenolol, alprenolol, betaxolol, carvedilol, metoprolol, labetalol, pindolol, sotalol, etc, except timolol and penbutolol are used as single l-isomer. However, it has been demonstrated that d,l- and d-propranolol can inhibit the conversion of thyroxin (T4) to triiodothyronin (T3), contrary to its l-form (19-21). Therefore, single d-propranolol might be used as a specific drug without β-blocking effects to reduce plasma concentrations of T3 particularly in patients suffering from hyperthyroidism in which racemic propranolol cannot be administered because of contraindications for β-blocking drugs (16). It is to know that for a racemic drug, each enantiomer possesses its own pharmacological activities that can be null, similar, different or opposite.

Many calcium channel antagonists are used under racemic form such as verapamil, nicardipine, nimodipine, nisoldipine, felodipine, mandipine etc, except diltiazem is a diastereoisomer with two pairs of enantiomers. For example, the pharmacological potency of S(-)-verapamil is 10-20 times greater than its R(+)-antipode in terms of negative chromotropic effect on AV conduction and vasodilatator in man and animals (22, 23). On the other hand, verapamil has another possible application in cancer chemotherapy as a modifier of multidrug resistance. Unfortunately, for this purpose, verapamil must be used at high concentrations leading to high cardiotoxicity. However, it was later found that R(+)-verapamil has far less cardiotoxicity than S(-)-verapamil. Therefore, the R-enantiomer would be preferable as a modifier of multidrug resistance in cancer chemotherapy, while the S-enantiomer or the racemate would be preferable as a calcium channel blocker for cardiovascular therapy (24).

All ACE inhibitors such as captopril, benazepril, enalapril, idapril are chiral compounds under diastereoisomeric form and most of them are marketed as single isomer. Valsartan, an angiotensin II receptor antagonist, is used as a single S-enantiomer and the activity of the R-enantiomer is clearly lower than the S-enantiomer (25).

Albuterol (salbutamol), salmeterol and terbutaline are sympathomimetic drug-selective β_2_-adrenoceptor agonists mainly used as bronchodilators in the treatment of asthma. They are longtime marketed as racemate. Pharmacologically, only their l-isomer or R (-)-isomer is effective and the other inactive d-or S (+)-isomer may be responsible for the occasional unpleasant side-effects associated with the drug. The Food and Drug Administration recently approved a chiral switch drug, levalbuterol (the pure l-isomer of albuterol) as a preservative-free nebulizer solution. However, some clinical studies recently reported that it is neither safer nor more effective than a same dose of racemic albuterol. In contrast, levalbuterol may cost as much as 5 times more than its racemate (26-27).

In neurology and psychiatry, many pharmaceuticals used are chiral compounds and most of them are marketed as racemates. Hypnotics such as hexobarbital, secobarbital, mephobarbital, pentobarbital, thiopental, thiohexital are racemic compounds and overall, only l-isomer is hypnotic or sedative, the other is either inactive or excitative. For example, S(-)-secobarbital is more potent as anesthetics than R(+)-secobarbital i.e. it causes a smoother more rapid anesthetic effect (13, 28). Ketamine is an intravenous anesthetic. The (+)-isomer is more potent and less toxic than its (-)-antipode, but unfortunately, ketamine is still used as racemic drug (5, 10). Isoflurane is an inhalational general anesthetic widely used in surgical operations as a racemic mixture of its two optical isomers. The (+) isomer of isoflurane is more effective than the (-) isomer at inhibiting currents induced by the bath application of acetylcholine (24). In the treatment of depression, S (+)-citalopram is over 100-fold more potent as a selective serotonin reuptake inhibitor than R (-)-enantiomer (3).

Methadone, a central-acting analgesic with high affinity for μ-opiod receptors, has been used to treat opiate dependence and cancer pain. Methadone is a chiral synthetic compound used in therapy under racemic mixture. In humans, R (-)-methadone is about [25-50] fold more potent as an analgesic than its S (+) antipode (3, 29, 30).

The list of racemic drugs with one eutomer is long. It includes anticonvulsants such as mephenytoine, ethosuximide; antiarrhythmics and local anesthetics such as propafenone, disopyramide, prilocaine, tocainide; antibiotics such as ofloxacin, moxalactam; anticoagulants such as warfarine, acenocoumarol; antihistaminics such as terfenadine, loratadine; antihyperlipidemic such as atorvastatin; psychostimulants such as amphetamine, metamphetamine; proton pump inhibitors such as omeprazole, pantoprazole, lansoprazole, etc (15, 31-32). Some of these racemates recently undergo chiral switch to single enantiomer such as levofloxacin (from ofloxacin), levalbuterol (from albuterol), escitazolam (from citalopram), esomeprazole (from omeprazole), dexketoprophen (from ketoprophen), dexmethylphenidate (from methylphenidate), etc.

### Group 2. Racemic drugs with equally bioactive enantiomers

There are only some racemic drugs that could belong to this group such as cyclophosphamide (antineoplastic), flecainide (antiarrhythmic), fluoxetine (antidepressant) (15).

### Group 3. Racemic drugs with chiral inversion

There are two kinds of drug chiral inversion: unidirectional and bidirectional inversion (31).

Unidirectional enzyme mediated inversion was previously described only with 2-arylpropionate nonsteroidal anti-inflammatory drugs (NSAID), namely ibuprofen, ketoprofen, fenprofen, benoxaprophen, etc. For this group, only S-enantiomer is active i.e. has an analgesic and anti-inflammatory effect. For example, S-ibuprofen is over 100-fold more potent as an inhibitor of cyclooxygenase I than (R)-ibuprofen. In the body, only inactive R-enantiomer can undergo chiral inversion by hepatic enzymes into the active S-enantiomer and not vice-versa (9, 31).

Bidirectional chiral inversion or racemization should be represented by 3-hydroxy-benzodiazepines (oxazepam, lorazepam, temazepam) and thalidomide in which R and S enantiomer can racemize *in vitro* by aqueous solution. However, *in vivo* this phenomenon could occur with thalidomide, but not with hydroxyl-benzodiazepines because of the differences in substituents around their chiral carbon. Some authors (33) have found for the first time the difference in R- and S-oxazepam concentrations in treated rabbit serum. They explained that the chiral inversion by tautomerization of oxazepam cannot occur *in vivo* because each enantiomer is transported by protein (albumin) with different affinity. The binding affinities of the enantiomers to albumin may inhibit the attack of hydroxyl ions (water) and thus retard the epimerization and racemization *in vivo*. Therefore, R- and S-oxazepam concentrations can be found different in the serum of these treated rabbits. On the other hand, He *et al* (34) have also demonstrated that the *in vitro* chiral inversion of these benzodiazepine enantiomers was temperature-dependent and was inhibited by lowering temperature of aqueous solution to about 10°C (33-34). The S (+)-oxazepam enantiomer is 100-200 fold more potent as a tranquilizer and sedative than R (-)-oxazepam (35).

Thalidomide is a former racemic sedative withdrawn from the market in the 1960s due to severe teratogenic effects (phocomelia, amelia). However, there is renewed interest in restricted use of thalidomide because of its immunomodulatory (36), anti-angiogenic, and anti-inflammatory effects (15) Moreover, it strongly inhibits the tumor necrosis factor α (TNF-α). Thalidomide gave spectacular results in the treatment of erythema nodosum leprosum, aptosis, Behcet’s syndrome and has been assayed for organ transplantation, some autoimmune diseases such as chronic lupus erythematosus, rheumatoid arthritis, some forms of cancer, etc (15, 36). Single thalidomide enantiomers and its derivative, N-hydroxythalidomide, were also synthetized by asymmetric technique in order to study their individual biological and chemical activities (37, 38). It seems that a multitude of its pharmacological activities could be due not only to the mother molecule but also to its numerous chiral and achiral metabolites. Because of this *in vivo* interconversion of thalidomide, it is difficult to determine exactly the pharmacological effect of each enantiomer.

The main pharmacological potency observed from two isomers of some current racemic drugs is gathered in the Table 1.

**Table 1:** Comparison of isomer potency of some racemic drugs (l=levorotary, d=dextrorotary)

Main pharmacological effects of drugs	Isomer potency

b -Adrenoreceptor blocking drugs (b -blockers): propranolol, acebutolol, atenolol, alprenolol, betaxolol, carvedilol, metoprolol, labetalol, pindolol, sotalol, etc,	l > d (d = inactive)
	Ex: S(-)-propranolol > R(+)-propranolol
Calcium channel antagonists: verapamil, nicardipine, nimodipine, nisoldipine, felodipine, mandipine etc,	l > d
	Ex: S(-)-verapamil > R(+)-verapamil
β2-Adrenoceptor agonists: Bronchodilators: Albuterol (salbutamol), salmeterol and terbutaline	l > d (d = inactive)
	Ex: R(-)-albuterol > S(+)- albuterol
Hypnotics, Sedatives: hexobarbital, secobarbital, mephobarbital, pentobarbital, thiopental, thiohexital	l > d
	Ex: S(-)-secobarbital > R-(+)secobarbital
Anesthetics: Ketamine, isoflurane	d > l (l = inactive)
	Ex: S(+)-ketamine > R(-)-ketamine
	S(+)-isoflurane > R(-)-isoflurane
Central-acting analgesic (μ-opiod receptors): Methadone	Ex: R(-)-methadone > S(+)-methadone
Analgesics, Anti-inflammatory : (NSAID): ibuprofen, ketoprofen, benoxaprophen, fenprofen, etc.	d > l
	Ex: S(+)-ibuprofen > R(-)-ibuprofen
Tranquilizers: 3-hydroxy-benzodiazepines: oxazepam, lorazepam, temazepam	d > l (l = inactive)
	Ex: S(+)-oxazepam > R(-)- oxazepam

## TOXICOLOGY

Since there are frequently large pharmacodynamic and pharmacokinetic differences between enantiomers, it is not surprising that enantiomers may result in stereoselective toxicity. In toxicology, the different toxic effects of chiral drugs can reside either in one enantiomer only or in both ones. The toxicological properties in a pair of enantiomers can be identical or entirely different. They can reside in the pharmacologically active enantiomer or in the inactive one (9, 12, 15, 25, 32, 40, 43).

Some following drugs are marketed as single enantiomer solely because their toxicities reside almost in one of their two enantiomers. Dopa or dihydroxy-3,4 phenylalanine is a precursor of dopamine that is effective in the treatment of Parkinson disease. Dopa was used under racemic form: d,l- dopa, but owing to the grave toxicity (agranulocytosis) of d-isomer, therefore, only levoratory form called L-Dopa is actually used in therapeutics. Tetramisole is a nematocide, first used under racemic form. Because of numerous side-effects (vertigo, headache, vomiting, abdominal pain) mainly due to d-isomer, therefore, only l-isomer called levamisole is now used in medicine. Actually, some racemic drugs cited in the chapter of pharmacology are transformed by chiral switch into their single active isomer because one of two isomer has side-effects and no pharmacological activity (9, 14, 15, 41, 43). However, a number of chiral drugs are still marketed under racemic form because either their chiral separation is difficult, or their pharmacologic and toxic effects reside in the same enantiomer or their high cost production (9, 14, 15, 41). Toxicity of chiral drugs like ketamine (anesthetic), penicillamine (chelating agent), ethambutol (antitubercular agent) reside exclusively in their distomer. For example, only R (-)-ketamine (distomer) is responsible for agitation, hallucination, restlesness, in contrast to S (+)-ketamine (eutomer) (40). However, secobarbital enantiomers are equipotent as anticonvulsants, but the S(-)-isomer is a more potent anesthetic and is also more toxic than the R(+)-isomer (40). In the case of cyclophosphamide, its two isomers exert the same toxicity (42, 43). For thalidomide, theoretically, only the inactive S(-)-isomer is teratogenic, but practically, both isomers are genotoxic because of its *in vivo* interconversion and of its species-dependence (42, 43). Tests with mice in 1961 suggested that only one enantiomer was teratogenic while the other possessed the therapeutic activity. Unfortunately, subsequent test with rabbits showed that both enantiomers had both teratogenicity. The S-isomer (in contrast to the R-isomer) has been linked to thalidomide’s teratogenic effects. However, attempts to formulate the R-isomer have not solved the problem of teratogenicity, as the two isomers are readily interconvertible *in vivo* (14, 39). Moreover, toxicity of thalidomide could be due to its numerous chiral and achiral metabolites of which pharmacological and toxicological studies remain very scarce.

## PHARMACOKINETICS AND METABOLISM

The processes of absorption, distribution, elimination and metabolism are crucial determinants of drug action and can assume equal relevance to the actual biological effect of the drug at its receptor site. The potential for discrimination between enantiomers at each of these stages is therefore important and emphasizes the need for stereo-pharmacokinetic studies and stereospecific drug assays (44). Indeed, numerous studies have demonstrated that stereoisomers of a chiral drug often exhibited pronounced differences in their pharmacokinetic and metabolic profiles both quantitatively and qualitatively (45-47).

According to Mehvar *et al*. (48), many antiarrhythmic drugs are marketed as racemates such as disopyramide, encainide, flecainide, mexiletine, propafenone, tocainide, etc. The absorption of chiral antiarrhythmics appears to be nonstereoselective. However, their distribution, metabolism and renal excretion usually favour one enantiomer versus the other. In terms of distribution, plasma protein binding is stereoselective for most of these drugs, resulting in up to two-fold differences between the enantiomers in their unbound fractions in plasma and volume of distribution. Hepatic metabolism plays a significant role in the elimination of these antiarrhythmics. Additionally, in most cases, significant stereoselectivity is observed in different pathways of metabolism of these drugs. Therefore, it is not surprising that a wide interindividual variability exists in the metabolism of these drugs. Overall, substantial stereoselectivity has been observed in both the pharmacokinetics and pharmacodynamics of chiral antiarrhythmic agents. Because the effects of these drugs are related to their plasma concentrations, this information is of special clinical relevance (48).

As reported by Stoschitzky *et al* (16), there are marked pharmacokinetic differences between the d- and l-enantiomers of most β-blockers, particularly under exercise and when extensive and poor metabolisers are compared. Plasma concentrations of these d and l-enantiomers usually differ significantly and in wide ranges when the racemic mixture is administered orally or intravenously. Mehvar *et al* (49) also reported that the β-blockers are quite diverse in pharmacokinetic profile, as they display a high range of values in plasma protein binding, in percent of drug eliminated by metabolism or unchanged in the urine, and in hepatic extraction ratio. With respect to plasma concentrations attained after oral or intravenous dosing, in most cases the enantiomers of the β-blockers show only a modest degree of stereoselectivity. However, the relative magnitude of the concentrations of the enantiomers in plasma is not constant in all situations and varies from drug to drug. Further, various factors related to the drug (e.g., dosing rate or enantiomer-enantiomer interaction) or the patient (e.g., racial background, cardiovascular function, or the patient metabolic phenotype) may affect the stereospecific pharmacokinetics and pharmacodynamics of β-blockers (49). In another study (50) the pharmacokinetic profile of propranolol enatiomers and their enantiomer sulfate and glucuronide metabolites, in human serum and urine showed that the S/R ratios of mother molecule in serum and urine were about 1.4, of sulfate conjugate about 2 and of major glucuronide metabolite about 3. But variation of the ratios S(-)- active/R(+)-inactive isomers between individuals was also observed (50).

Methadone, a central-acting analgesic, used in the treatment of opiate addicts is a racemate with predominantly active R(+)-isomer. In humans, the ratios of R/S methadone levels varied from 0.6 to 2.0 in serum and 1.2-2.0 in urine because of inter-individual differences in the pharmacokinetics of methadone enantiomers (3, 30, 51, 52). Methadone is mainly metabolized by hepatic cytochrome P-450 3A4 and secondary by P-450 2D6 to a major methadone metabolite, EDDP, (2-ethylidene-1,5-dimethyl-3,3-diphenylpyrrolidine). In human urine, EDDP levels were always higher (about 1.5-5 fold) than those of mother molecule because of its polarity, however its concentrations were undetectable in human saliva and always lower than those of methadone in serum (53).

Although the *in vitro* chiral inversion of 3-hydroxy-benzodiazepines in aqueous solution, some authors (33) were the first to demonstrate the difference in serum concentrations of two oxazepam enantiomers, with predominance of the active S (+) form in rabbits treated with pharmacological and toxic dosages, while the inactive R (-) antipode was higher in intoxicated rabbits under antidote treatment with flumazenil. Because of the high binding of oxazepam enatiomers to protein (97%), the hydroxyl ions of water in body cannot racemize each enantiomer. Differences in the binding affinities of each oxazepam enantiomer to protein may account for the selectivity of the drug and could explain the variation of the ratios R/S enantiomer concentrations in serum (33).

An understanding of the stereospecific pharmacokinetics of all chiral drugs may help clinicians to interpret and predict differences among patients in pharmacologic responses to the racemic drugs and to adjust their dosage for each patient.

## ENANTIOSELECTIVE ANTIBODIES TO CHIRAL DRUGS

As many target organs can distinguish the two enantiomers of a chiral drug, it is not surprising that the immune system may recognize them in the same manner by producing selectively and specifically each corresponding enantiomer antibody. Such enantioselective antibody can distinguish even minor differences in composition or configuration of a chiral drug. As drugs are small molecules called haptens, they need to be conjugated to a support or to a carrier molecule (protein) by forming an immunogen so that the immune system (B cells) could produce corresponding antibody (54, 55). The conjugation of a hapten such as an enantiomer with a protein is a delicate and difficult step of the immunogen preparation. The specificity and sensitivity of an antibody against an enantiomer drug mainly depends on the hapten preparation and the conjugation technique. According to Sahui-Gnassi *et al*. (56) and Chikki-Chorfi *et al*. (58) for the obtention of a specific antibody to a chiral compound, it is necessary to keep intact the carbon asymmetry i.e. no direct linking of hapten with protein carrier at the chiral center. In the literature, some enantioselective antibodies have been successfully used to separate enantiomers, such as antibodies to propranolol enantiomers (56) methadone enantiomers (58) amphetamine enantiomers (55) warfarin enantiomers (55) etc. However, they are still scarce in comparison with numerous antibodies to achiral drugs. The application of enantioselective antibodies to chiral drugs plays a key role in biochemistry. They can be used as a specific reagent not only for immunoassays such as radioimmunoassay (55) enzymatic immunoassay (57) but also for histologic immunoassay, immunoaffinity chromatography, immunoextraction of chiral drugs, liquid chromatography using antibodies as chiral selectors, etc. These immunoassays can be applied to pharmacokinetics, drug therapeutic monitoring, toxicological diagnostic, drug pharmacological assessment, identification of drug fixed on target organ, etc.

## MECHANISM OF BIOLOGICAL ACTIVITY

**Figure 2:**
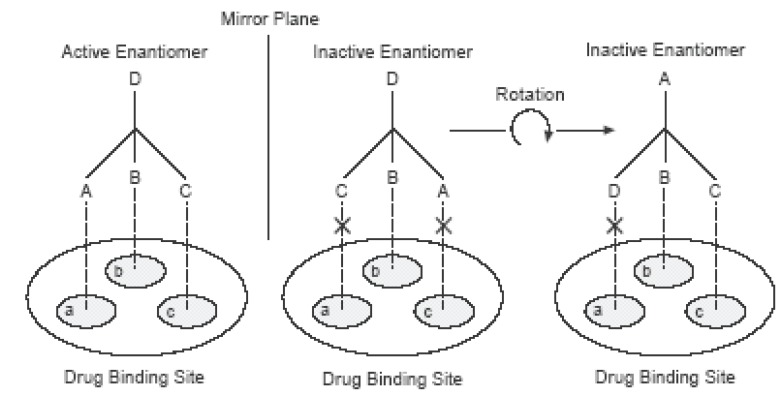
Easson-Stedman hypothetical interaction between the two enantiomers of a racemic drug with a receptor at the drug binding sites. The three substituents A, B, C of the active enantiomer (left) can interact with three binding sites a, b, c of a receptor by forming three contacts Aa, Bb and Cc, whereas the inactive enantiomer (right) cannot because the contact is insufficient. Note: This figure is in the publication of McConalthy and Owens (47).

The enantiomers of a chiral drug may vary in their interactions with chiral environments such as enzymes, proteins, receptors, etc of the body. These variations may lead to differences in biological activities such as pharmacology, pharmacokinetics, metabolism, toxicity, immune response etc. Indeed, biological systems can recognize the two enantiomers as two different substances, and their interaction each other will therefore elicit different responses. But, why do enantiomers have different biological activities? The reason for chiral recognition by drug receptors is a three-point interaction of the drug with the receptor site proposed by Easson and Stedman (44, 47, 59). The difference between two enantiomers of a drug with its receptor is illustrated in Figure 2 (published by McConathy and Owens (47) using a hypothetical interaction between a chiral drug and its chiral binding site (47). In this case, one enantiomer is biologically active while the other enantiomer is not. The substituents of the active enantiomer drug labeled A, B, and C must interact with the corresponding regions of the binding site labeled a, b, and c of the receptor in order to have an alignment Aa, Bb, Cc. In this case, this fitting interaction can produce an active biological effect. In contrast, the inactive enantiomer cannot bind in the same way with its receptor when it rotates in space, consequently, there is no active response (44, 47). To resume this hypothesis, the attachment of an enantiomer to the chiral receptor is similar to a hand fitting into a glove or to a key into a lock. Indeed, a right hand can only fit into a right hand glove, so a particular enantiomer can only fit into a receptor site having the complimentary shape. The other enantiomer will not fit, like a right hand in a left glove, but may fit into a receptor site elsewhere in the body and cause an eventual unwanted or toxic effect. On the other hand, enantiomers can show different chemical behaviour due to different chiral discrimination by diastereomeric formations with a chiral environment (9, 12, 44, 47, 59).

## CHIRAL SEPARATION

Chiral separation, also called chiral resolution, is a procedure used to separe the two isomers of a racemic compound in pharmaceutical industry as well as in clinical analysis. Various methodologies used for chiral separation on both analytical and preparative scales will be described below.

### In pharmaceutical industry

During chemical synthesis, many drugs obtained can be racemized *in situ* by a variety of chemical reactions, even the procedure used has started with pure enantiomeric reagents. In industry, two main categories of techniques are often applied for chiral resolution: the classical methods and the modern technologies (60, 61).

For the classical approach, the most widely used technique is the resolution by diastereomeric salt formation. In this strategy, an acid-base reaction is involved between a racemic drug and a pure single enantiomer called resolving agent. This reaction leads to the formation of two diastereomeric salts that now have different physical and chemical properties. These two diastereomers obtained can be easily separated either by crystallization or by filtration if one is soluble and the other is insoluble. Finally, the salt is decomposed by treatment with either acid or base, then the pure enantiomer is obtained (44, 60). The two diastereomers formed can also be separated by classical achiral liquid chromatography. This method has been used in the resolution of -methyl-L-dopa, asparagine and glutamic acid (44). Another classical approach is the enzymatic or kinetic resolution. In this methodology, resolution is achieved by means of biochemical process that destroys one enantiomeric form. Certain microorganisms such as yeasts, molds, bacteria can only degrade one of two isomers of a racemate by enzymatic assimilation, the other which is not digested remains in solution, then it is isolated (60, 61). Enzymatic resolution has been used in the preparation of lotrafiban (benzodiazepine), levofoxacin (antibacterial drug), and S-naproxen (antiinflammatory drug) (44).

For the modern technologies, preparative high-performance liquid chromatography (HPLC) is the method of choice for the enantiomer separation. Chiral HPLC has proven to be one of the best methods for the direct separation and analysis of enantiomers. In chromatographic methods, two techniques are used: indirect and direct. The indirect HPLC involves derivatization of samples with a chiral derivatization reagent i.e. a pure single enantiomer, resulting in the formation of two diastereomers which can be separated by a classical reversed-phase column (62). This indirect HPLC method is rarely used in industry, but frequently performed in biological analysis because of its high sensitivity. On the other hand, the direct HPLC utilizes the chiral selector either in chiral stationary phases (CSPs) or in the mobile phase called chiral mobile phase additives (CMPA) (60, 63, 64). The last technique is rarely used in industry because of its high cost and low efficiency. Direct chiral separations using CSPs are more widely used and are more predictable, in mechanistic terms, than those using chiral additives in the mobile phase. Among a hundred HPLC CSPs commercially available, only some types of chiral sorbents following are presently the most widely used for preparative HPLC in industry: carbohydrate (cellulose, amylose), polyacrilamide, diallyltartardiamide, Pirkle phases, chirobiotic phases (vancomycin, teicoplanin) (64). However, there is no single CSP that can be considered universal, i.e., has the ability to separate all classes of racemic compounds. Choosing the right column for the enantioseparation of a racemic compound is difficult. The decision relies mostly on empirical data and experience (60). However, the understanding of the recogntion mechanisms of chiral selectors with enantiomers can help the chromatographists to resolve some problems of resolution and to economize time-consuming. According to Aboun-Enein and Ali (60) all chiral selectors provide a chiral surface to enantiomers, which form with the selectors temporary complexes, having different bonding energies. The enantiomers differ in their binding energies because they fit differently into the chiral selector structures. Consequently, the two enantiomers can be eluted at different times by the mobile phase and then separately collected. Brieftly, in general, the recognition mechanism on a chiral selector is based on a key-and-lock arrangement (60). However, many other factors such as mobile phase composition (pH, electrolytes, solvent nature), size and length of column, temperature etc also play a key role for chiral resolution.

Besides the direct chiral HPLC, a new technique called simulated moving bed (SMB) chromatography is recently developed for industry. As reported by Burke and Henderson (44) the basic concept of SMB technology is the continuous countercurrent movement of stationary and mobile phases in which the movement of a stationary phase is simulated. The small particles in this component are packed into single columns and connected to form a circle. Four external valves allow the addition and subtraction of feed and effluent. The mobile phase is pumped through the circle and when it passes the stationary phase a slight separation occurs, the less absorbable compound running in front and the more absorbable compound staying behind. When steady state is reached, the system can be operated continuously. If all flow rates and the shift time are determined correctly, raffinate and extract fractions can be withdrawn in high purity (44, 65). An example of a pharmaceutical compound separated by SMB chromatography is tramadol (44). The scheme of the SMB chromatography is described in the article of Johannsen *et al* (65). The SMB procedure allows to reduce solvent consumption, and consequently may lower the production cost.

To avoid the racemization *in situ* during chiral drug preparation, an asymmetry synthesis using chiral catalysts has been developed by W.S. Knowles, R. Noyori and K.B. Sharpless, the Nobel Prize in chemistry 2001 (6, 66). Most of the available asymmetric chemical catalysts are organometal types including transition metals such as titanium, and noble metals such as osmium, palladium, and rhodium. Chiral catalysts are like enzymes in that both have a high degree of specificity. They allow stereospecific reactions to take place and therefore avoid the formation of racemates. Chiral chemical catalysts are hardier than enzymes, and tolerate higher temperatures. However, the use of chiral chemical catalysts is usually costly. L-Dopa (anti-Parkinson agent), naproxen (anti-inflammatory drug) are some examples of single enantiomer drugs produced by this catalytic asymmetric synthesis (24, 66, 67).

### In clinical analysis

The analyses of chiral drugs in biological fluids are much more difficult than their quantifications and separations in industry although they can use the same physical techniques. Because of the complexity of biological samples and also of the low concentrations of drugs in serum and urine from μg to ng/ml, the determination of these drugs requires first an extraction with high recovery from biological samples, then an analytical technique with high sensitivity, precision and reproducibility.

Extraction. The extraction of drugs and toxics in biological matrices for physical chemical analysis (HPLC, GC, MS), a key step of bioanalysis, is used to clean the samples by removing proteins and other interfering biological compounds before analysis. For the extraction of a pair of enantiomer drug from biological samples, two techniques are used: liquid-liquid extraction and solid-phase extraction (SPE). For the first technique, the choice of extraction solvent depends on the polarity of the chiral drug. In most cases, a buffer with pH2-10 is usually added into the sample before solvent extraction in order to liberate drug from protein. As the two enantiomers have the same chemical and physical properties, in general, the recoveries of two enantiomers are the same for the control samples. The classical liquid extraction is in general time-consuming, but it is cheaper and sometimes it can give high recovery and clean extract. However, some new liquid-phase microextraction techniques recently cited in the literature seem simple and rapid (68). For the solid-phase extraction technique, two kind of sorbents are used: classical reversed-phase silica-bonded with C18 and recent polymeric sorbent. The last extraction technique with polymeric sorbent showed a great advantage about recovery and universal use in comparison with classical reversed-phase cartridge as He *et al* (69) have recently published.

### Analytical methods

For the bioanalysis of racemic drugs, two kinds of analytical methods have been developed: the physical methods and the enantioselective immunoassays.

Chiral analysis by physical methods. In the first group, chiral chromatography including high performance liquid chromatography (HPLC), gas chromatography (GC), supercritical fluid chromatography (SFC) and capillary electrophoresis (CE) is most readily accomplished for the enantiomer resolution. HPLC is the most widely used of the four methods. As in industry, two HPLC techniques are used: indirect and direct. In contrast to industry, the indirect HPLC using chiral derivatization reagent with the formation of two diastereomers is frequently performed in bioanalysis because of its high sensitivity. However, this indirect technique requires a functional group in the analyte (drug) e.g. amine, hydroxyl, carboxyl, carbonyl and thiol. A chiral derivating reagent (a pure single enantiomer) added in the sample will react with these functions to form two diastereomers that can be separated by a classical reversed-phase column (C18 or C8) (62). For example, propranolol (β-blocker), perhexiline (antianginal agent) have been determined by this indirect HPLC with fluorescent detector (50, 70). Because of the limitation of the indirect HPLC, direct chiral separations using chiral stationary phases CSPs are the most used because of its simplicity and its rapidity. Today, nearly a hundred CSPs have been developed and are recently marketed, but some types of chiral HPLC columns following are the most used in bioanalysis: cyclodextrine and its derivatives, carbohydrate (cellulose, amylose), Pirkle phases, chirobiotic phases. For example, many racemic drugs are well resolved with cyclodextrine and its derivative phases (Cyclobond I^®^, Cyclobond I-2000 RSP^®^, Cyclobond I-2000 SP^®^) such as propranolol (71) methadone (30, 69) 3-hydroxybenzodiazepam (oxazepam, lorazepam, temazepam) (33, 34) Cellulose and its derivatives such as Chiralcel OJ^®^ with pure ethanol as mobile phase can separate thalidomide enantiomers (37) tetrahydropalmatine enantiomers (synthetic alkaloid of carydalis used as antiarrhytmic and antihypertensive) (72). Amylose and its derivatives such as Chiralcel OD^®^ is used to separate donepezil enantiomers (anti-acetylcholinesterase for the treatment of Alzheimer disease) (73). Chiralpak AD^®^ to resolve metoprolol enantiomers (β-blocker) (74) or other racemic drugs with polar organic solvent chromatography (75). Antibiotics or chirobiotic phases such as vancomycin-CSP or eremomycin-CSP can also separate thalidomide and amino acids, respectively (76, 77). Amide derivative XAD-4 CSP was used for chiral chromatographic separation of many β-blockers (78). Liquid chromatography-mass spectrometry (LC-MS), gas chromatography-mass spectrometry (GC-MS) and capillary electrophoresis (CE) are other physical methods for the separation of numerous chiral pharmaceuticals (79-81). Unfortunately, until today, there is no single CSP that can resolve all classes of racemic compounds in bioanalysis, contrary to achiral reversed- phase C18 or C8. The choice of a chiral column is in general examined on the interaction mechanism between CSP and chiral analyte (82).

Enantioselective immunoassays. In contrast to physical methods, immunoassays do not require a preliminary extraction of biosample before analysis. Moreover, their sensitivity is higher and a small amount of sample about some μl to ten μl is sufficient. If the determinations of achiral drugs by immunoassays such as EMIT^®^, FPIA or TDx^®^ are widely used in therapeutic drug monitoring, the use of enantioselective immunoassays is still very scarce in clinic. Some techniques such as radioimmunoassays or enzymo-immunoassays (ELISA) used for the determination of some enantiomer drugs (propranolol, methadone, amphetamine, warfarin, atropine, pentobarbital) were developed, but still on experimental scale (55-58). Moreover, these chiral immuoassays are still not automatized, contrary to classical immunoassays (EMIT^®^, FPIA or TDx^®^ etc).

## DISCUSSIONS

In recent years, as patent on a successful drug nears expiration, pharmaceutical manufacturers have sometimes marketed a single stereoisomer of the old racemic drug as a new drug (chiral switch), often with claims of greater activity, less toxicity or both. Many currently marketed drugs are racemic mixtures of stereoisomers. They may be enantiomers, which are non-superimposable mirror images, or geometric isomers, which are not mirror images, but in either case stereoisomers can differ markedly from each other in bioactivity and pharmacokinetics. The FDA now requires manufacturers to identify and characterize each individual isomer of a new racemic mixture. However, the differences between stereoisomers may not be clinically significant. The levofloxacin, S(-) isomer of ofloxacin, has an important clinical advantage over racemic ofloxacin, but for some other stereoisomers marketed recently as the patent was expiring on the original racemic mixture (such as esomeprazole, levalbuterol, dexmethylphenidate and escitalopram), no such advantage has been clinically demonstrated.

Accounting for the growing development of chiral drugs as racemate and single enantiomer worldwide, it is primordial to promote the chiral separation and its development because this operation plays a key role not only in pharmaceutical industry but also in clinical therapeutics. Nowadays, many drugs are still used as racemates with their side-effects, this problem is probably due not only to the difficulty in chiral separation technique but also to the production costs. If a new separation method for chiral drugs will be developed with its large application scale and low cost, the number of racemic drugs could diminish significantly. The direct production of a single drug enantiomer by asymmetric synthesis is useful when its other antipode is found toxic or entirely inactive. However, for the drug discovery process, the obtention of a racemate could give triple informations about the drug to be explored i.e. informations about the racemate and also about the two single enantiomers. Theoretically, the use of a single isomer is ideal, but practically, the decision must be taken after long clinical observation between racemate and single enantiomer actions. In some therapeutic cases, the use of a racemate is more helpful than that of each single isomer because of the complementary effects of each other. Therefore, preparative and analytical HPLC are very useful at the experimental step of drug discovery and also are an invaluable tool for the searcher. The ultimate choice of the separation technique either physical or chemical depends on the nature of each drug to be produced and also on the quantity, the time and the cost of the production.

Our body is a great factory of chiral selectors, and could well distinguish the stereoform of a chiral drug, we can ask if it is interesting to transform some old achiral drug into its chiral derivative in order to discover some unexpected pharmacological effect. Thalidomide is a chiral drug with a multitude of pharmacological activities, maybe some of them could be given by one or more of its numerous chiral metabolites.

In clinical therapeutics, the use of a chiral assay is still not universally performed. Use of a non-stereoselective determination for a drug administered as a racemate may result in erroneous therapeutic interpretation. For example, for the same dosage of a racemic drug administered to two patients, a non-chiral assay can give the same racemate concentrations for both patients. But, the ratio of the active form and the inactive one can differ in two patients and thanks to the last results, the physician can correctly interpret the difference in clinical observation between them. Therefore, it is important to promote the automatization of some chiral techniques used in clinic such as chiral HPLC and enantioselective immunoassays. It is also helpful to inform their utility to doctors and pharmacists. Besides, drug dictionary and pharmacopeia have to mention the chiral form of a drug (i.e. racemate or enantiomer) as well as the pharmacological, pharmacokinetic and toxicological effects of each isomer. These informations will be a precious guide for all healthcare professionals.

## CONCLUSIONS

The chiral separation of racemic drugs is a necessary operation in pharmaceutical industry as well as in clinical therapeutics. Therefore, the development of new chiral separation techniques is and will be a topic subject in academic research as well as in industrial advance. However, the use of a single isomer must be seriously taken after long clinical assessments between racemate and single enantiomer actions because in some cases, racemates have more therapeutic advantages than single isomers. It is also important to give more informations about chiral drugs especially racemic form to healthcare professionals in order to help them for finding an optimal treatment and a right therapeutic control.
